# AAV Gene Therapy for MPS1-associated Corneal Blindness

**DOI:** 10.1038/srep22131

**Published:** 2016-02-22

**Authors:** Melisa Vance, Telmo Llanga, Will Bennett, Kenton Woodard, Giridhar Murlidharan, Neil Chungfat, Aravind Asokan, Brian Gilger, Joanne Kurtzberg, R. Jude Samulski, Matthew L. Hirsch

**Affiliations:** 1 Gene Therapy Center, University of North Carolina at Chapel Hill, NC, 27599, USA; 2 Department of Ophthalmology, University of North Carolina, Chapel Hill, NC, 27599, USA; 3 Department of Genetics, University of North Carolina, Chapel Hill, NC, 27599, USA; 4 Department of Pharmacology, University of North Carolina, Chapel Hill, NC, 27599, USA; 5 College of Veterinary Medicine, NCSU-CVM, Clinical Sciences, Raleigh, NC, USA; 6 Department of Pediatrics, Duke University, Durham, NC, 27710, USA.

## Abstract

Although cord blood transplantation has significantly extended the lifespan of mucopolysaccharidosis type 1 (MPS1) patients, over 95% manifest cornea clouding with about 50% progressing to blindness. As corneal transplants are met with high rejection rates in MPS1 children, there remains no treatment to prevent blindness or restore vision in MPS1 children. Since MPS1 is caused by mutations in *idua*, which encodes alpha-L-iduronidase, a gene addition strategy to prevent, and potentially reverse, MPS1-associated corneal blindness was investigated. Initially, a codon optimized *idua* cDNA expression cassette (opt-IDUA) was validated for IDUA production and function following adeno-associated virus (AAV) vector transduction of MPS1 patient fibroblasts. Then, an AAV serotype evaluation in human cornea explants identified an AAV8 and 9 chimeric capsid (8G9) as most efficient for transduction. AAV8G9-opt-IDUA administered to human corneas via intrastromal injection demonstrated widespread transduction, which included cells that naturally produce IDUA, and resulted in a >10-fold supraphysiological increase in IDUA activity. No significant apoptosis related to AAV vectors or IDUA was observed under any conditions in both human corneas and MPS1 patient fibroblasts. The collective preclinical data demonstrate safe and efficient IDUA delivery to human corneas, which may prevent and potentially reverse MPS1-associated cornea blindness.

Mucopolysaccharidosis1 (MPS1) is an autosomal recessive lysosomal storage disorder caused by null or nonsense mutations in the gene encoding alpha-L-iduronidase (IDUA), a ubiquitous intracellular and secreted enzyme that breaks down glycosaminoglycans (GAGs). In the absence of functional IDUA, GAGs accumulate in lysosomes and disrupt the normal intracellular trafficking of lipids, sugars, and proteins causing multisystem end organ damage. The incidence of MPS1 is approximately 1 in 100,000 and the disease is characterized by hepatosplenomegaly, cardiac insufficiency, bone and joint deformities, dwarfism, mental retardation and serious nervous system problems, which commonly result in death by 10 years of age. Additional symptoms include hearing loss, joint stiffness, and clouding of the cornea which results in loss of vision^1^.

Current MPS1 treatments include IDUA enzyme replacement therapy (ERT) (Aldurazyme) via intravenous injections, which has proven useful in reducing hepatosplenomegaly, and in improving myocardiac function, pulmonary symptoms and motility in MPS1 patients with mild disease. A more promising treatment relies on allogeneic hematopoietic stem cell transplantation (HSCT) which has been used for the past 2–3 decades in MPS1 patients. HSCT has proven successful at improving cognitive function, reducing hepatosplenomegaly, preventing ischemic cardiac disease and prolonging the patient’s lifespan, in some cases by decades. Clinical outcomes are best when myeloablative chemotherapy and cord blood donors are utilized, as it increases the extent of sustained donor chimerism. A third MPS1 treatment approach that is still under preclinical evaluation relies on an adeno-associated virus (AAV) IDUA gene addition strategy. Central nervous system (CNS) targeted AAV-IDUA gene therapy has been explored in murine, feline, and canine MPS1 models following administration via several routes including the carotid artery, intraparenchymal, intraventricular and intrathecal^2^,^3^,^4^. These gene therapy studies independently report histological, biochemical, and in particular cognitive improvements while, importantly, IDUA-related toxicity was not observed. However, ERT, HSCT and AAV CNS-targeted or systemic gene therapy exhibit a common deficiency; the inability to correct MPS1-associated maladies in privileged compartments including the joint and eye.

Regarding the ocular abnormalities, approximately 90% of MPS1 children lose vision due to corneal clouding, which has been attributed to the abnormal presence of vacuolated stroma cells^5^. Detailed analyses of MPS1 human corneas demonstrated an accumulation of chondroitin and dermatan sulphate GAGs, which alter the uniform distribution, organization, and size of collagen fibrils^6^,^7^. Cornea transplantation in MPS1 children has been used to address the corneal blindness, however the high rejection rate has, in recent years, discouraged this treatment as standard practice. On the contrary, isolated reports using HSCT have demonstrated the capacity to stabilize, improve, and in some cases restore corneal clarity^7^,^8^,^9^,^10^,^11^,^12^. These data strongly support our hypothesis that cornea abnormalities can be prevented or reversed in the presence of IDUA^8^.

Cornea targeted AAV gene therapy has been investigated in animal models primarily following 2 routes of administration; topical applications in wound healing assays and direct injection to the corneal stroma. Regarding topical applications, AAV serotype 9 (AAV9) was reported most efficient for stromal transduction, however, this was nearly entirely localized to the epithelial/stromal boundary^13^. Regarding AAV gene delivery following intrastromal injection into human cornea explants, it was observed that AAV8 was more efficient than AAV2 or AAV1 for stromal transduction, which encompassed multiple cell types including CD34 + keratocytes and macrophages^14^. Importantly, both of these routes of drug administration observed no deleterious consequences related to the AAV vector^13^,^14^,^15^.

The purpose of the present study is to explore AAV-mediated IDUA delivery as an effective treatment for MPS1 cornea clouding. Towards this end, we engineered an AAV vector cassette that efficiently restores IDUA to MPS1 patient fibroblasts in a dose-dependent manner. Normal, and supraphysiological, IDUA activity was demonstrated in both cellular lysates and in culture supernatants. Then, as MPS1 corneal disease has been attributed to stroma abnormalities^5^, the AAV serotypes reported most efficient for stromal transduction were evaluated in human corneas. The results demonstrate efficient transduction (AAV9 > AAV8), however, an AAV8/9 chimeric (8G9) capsid performed significantly better than either parental serotype. Using AAV8G9, IDUA was over-produced in the human corneal stroma with widespread distribution in multiple cell types, which included cells that naturally produce IDUA. Functional assays in human corneas demonstrated a 10-fold elevation of IDUA activity seven days post-injection without any indications of toxicity. Given the popularity of AAV gene therapy in ocular clinical trials, the soluble nature of IDUA, and the potential for compassionate use of AAV-IDUA in MPS1 children, our preclinical data herein provides encouraging results for a potential treatment of MPS1-associated corneal clouding.

## Results

**Figure 1:**
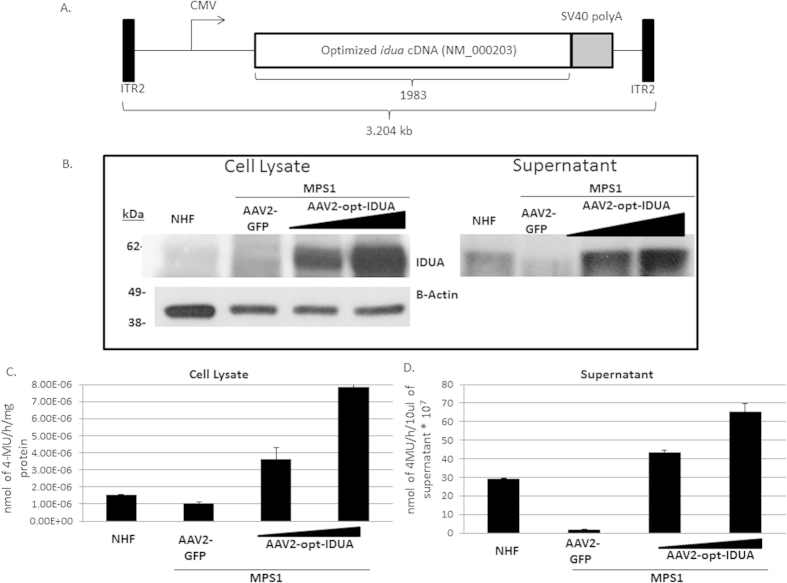
Restoration of IDUA activity in MPS1 patient fibroblasts by AAV gene therapy. (**A**) Schematic diagram of the AAV-IDUA optimized vector construct. (**B**) Cell lysates (Left panel) and supernatants (Right panel) from AAV2 infected and not-infected fibroblasts, normal human fibroblasts (NHF) or MPS1 fibroblasts, were analyzed for IDUA protein expression. Detection of β-actin was performed as a loading control of total protein in cell lysates. All the experiments were performed in triplicate. (**C**) Functional activity of IDUA protein was obtained for cell lysates and supernatants from AAV2 infected and not-infected fibroblasts, NHF or MPS1 fibroblasts. For cell lysates, the nmoles of 4-MU were normalized to one hour reaction and mg total protein. For cell supernatants, the nmoles of 5-MU were normalized to 10 μl of the sample. All the experiments were performed in triplicate. 4-MU, 4-methylumbelliferone; CMV, cytomegalovirus promoter; ITR, inverted terminal repeats; GFP, Green fluorescence protein.

To develop an AAV IDUA expression cassette, the human *idua* cDNA (NM_000203) was codon optimized for human production (opt-IDUA) and situated between the CMV promoter and the SV40 poly-adenylation sequence in an AAV inverted terminal repeat serotype 2 plasmid context (Fig. 1A). Production of IDUA protein by the pTR-CMV-opt-IDUA plasmid was confirmed, after performing transfection and Western Blot experiments with human embryonic kidney 293 cells and MPS1 patient fibroblasts (Fig. S1A,B). Additionally, IDUA protein levels from the codon optimized open reading frame were similar to that generated by wild type IDUA cDNA in both 293 cells and MPS1 patient fibroblasts (Fig. S1).

AAV2-opt-IDUA vectors were prepared as described^16^ and used for characterization in MPS1 patient derived fibroblasts. Immortalized normal human fibroblasts (NHF) served as the control cell line^17^. In dose escalation experiments, AAV2-opt-IDUA transduction of MPS1 fibroblasts resulted in increasing levels of IDUA restoration, in both cellular lysates and in the culture supernatant (Fig. 1B). In fact, as resting levels of IDUA in NHF are relatively low, a dose of 5,000 viral genomes/cell resulted in already supraphysiological levels in both cell lysates and in culture supernatants (Fig. 1B). Consistently, IDUA function was restored and elevated in transduced MPS1 patient fibroblasts with a 10-fold and 30-fold increases in cellular lysate and supernatant, respectively, at the highest investigated dose (Fig. 1C,D). Despite IDUA overproduction following AAV vector transduction, no toxicity was observed in patient fibroblasts at any dose using a dye exclusion assay (Fig. S2). Importantly, these results demonstrate the functionality and safety of AAV2-opt-IDUA in a MPS1 patient context.

**Figure 2:**
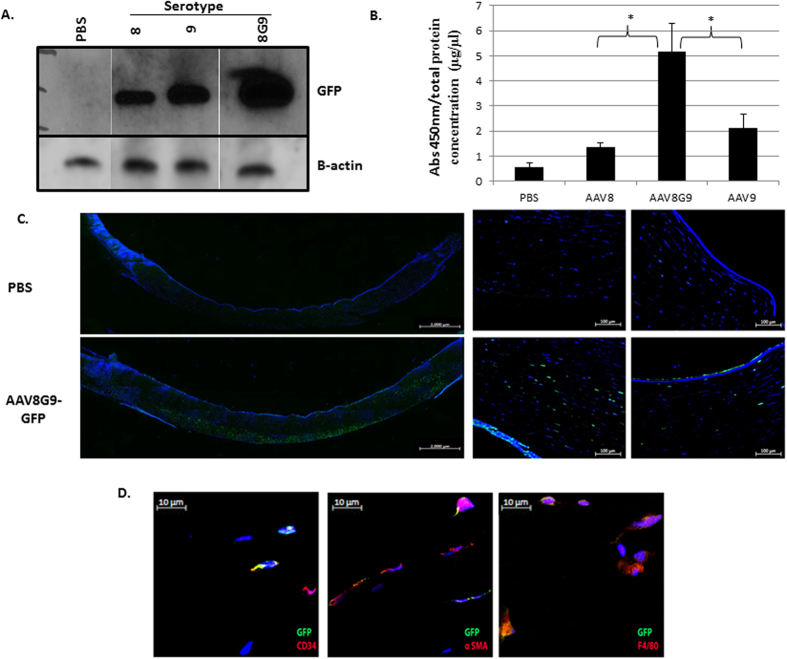
AAV capsid serotype evaluation in human cornea. (**A**) Human corneas were injected with a self-complementary CMV-GFP cassette encapsidated in AAV serotypes 8, 9, or the 8/9 chimeric 8G9. Seven days later Western blot was used to detect GFP. PBS corresponds to a human cornea injected with only PBS (vehicle control). Detection of β-actin was performed as a loading control. (**B**) Human cornea pieces were incubated with 1 × 10^10^ viral genomes of each serotype: 8, 9, or the chimeric 8G9, but as single stranded viruses. Seven days following viral infection, the tissues were collected and the protein lysates were obtained for quantification of GFP. Human corneas incubated with the medium but replacing the viral addition for PBS were used as negative controls (PBS). Absorbance measurements at 450nm were normalized to total protein concentration. (*corresponds to p ≤ 0.05 when performing T-tests comparing normalized absorbance 450nm values for AAV8G9 with AAV8 or AAV9; N = 5). (**C**) Single-strand AAV8G9-CMV-GFP was injected into the stroma of human corneas and harvested for histology 7 days later. Left- Immunofluorescence image showing the distribution of recombinant AAV8G9-GFP transduction across a human cornea section. Human corneas injected with PBS served as the negative control. Images were obtained with 10× objective and assembled by stitch processing. The scale bar is equal to 2000 μm. Right- Different areas of the same stained human cornea taken with 20× objective. The scale bar is equal to 100 μm. Hoechst was used for nuclei counterstain. (**D**) A representative section of the corneas in (**C**) stained with GFP (green) and the indicated cell marker (red). The scale bar is equal to 10 μm. Hoechst was used for nuclei counterstain.

An analysis of MPS1 patient corneas attributed stromal abnormalities as the cause of the corneal opacity that results in vision loss^5^. Therefore, direct administration of AAV-opt-IDUA should restore IDUA activity in the corneal stroma compartment of MPS1 patients, hopefully preventing or reversing the MPS1 phenotype. Previous reports demonstrated the utility of both AAV8^14^ and AAV9^13^ for human keratocyte transduction, one of the most prevalent cell types present in the cornea. As such, these capsids, carrying a self-complementary (sc) AAV-CMV-GFP genome, were evaluated in normal human corneal explants, which remain viable for weeks post-mortem. Preliminary experiments demonstrated that a volume of 50μl injected into the stroma was tolerated with minimal tissue distension and a distribution across ≈50% of the adult cornea using the vector vehicle containing india ink (Fig. S3). Therefore, AAV serotypes 8 or 9 containing a GFP reporter gene were administered to human cornea stroma by injection (1e^10^ vg in 50 ul) and analyzed seven days later by Western blotting. Given the reports of both 8 and 9 for cornea transduction, an AAV8/9 chimeric capsid (8G9), containing the galactose receptor of AAV9 engrafted on the AAV8 capsid was also investigated in the same manner^13^,^14^. Western blotting suggested that AAV8G9-GFP resulted in greater transduction than either parent serotype (Fig. 2A). In order to quantify the degree of viral transduction of the different serotypes on the human corneas, we performed a quantitative ELISA GFP assay (Fig. 2B). Single stranded versions of the AAV serotypes 8, 9 and 8G9 carrying the CMV-GFP cassette were used for cornea infection for seven days. After that time, viral transduction was determined according to the amount of GFP expressed by the infected cells. Statistical analysis revealed that AAV8G9 was able to infect the human cornea to a higher extent, compared to the transduction obtained by the parental capsids AAV8 or AAV9; p ≤ 0.05 for both cases. This analysis was performed with a total of five human corneas of mixed gender.

In order to determine the biodistribution of single-strand AAV8G9-GFP transduction (final volume 50 μl), GFP immunofluorescence was performed on human corneal cross sections. The results demonstrate a lateral spread of vector transduction across the cornea with penetration into the deeper stromal layers, including some endothelial cell transduction (Fig. 2C). Co-staining with CD34, a marker of keratocytes, with alpha-smooth muscle actin (alpha-SM), indicative of differentiated keratocytes, and with F4/80, a macrophage marker, demonstrated the corneal cell promiscuity for the AAV8G9 capsid (Fig. 2D).

**Figure 3:**
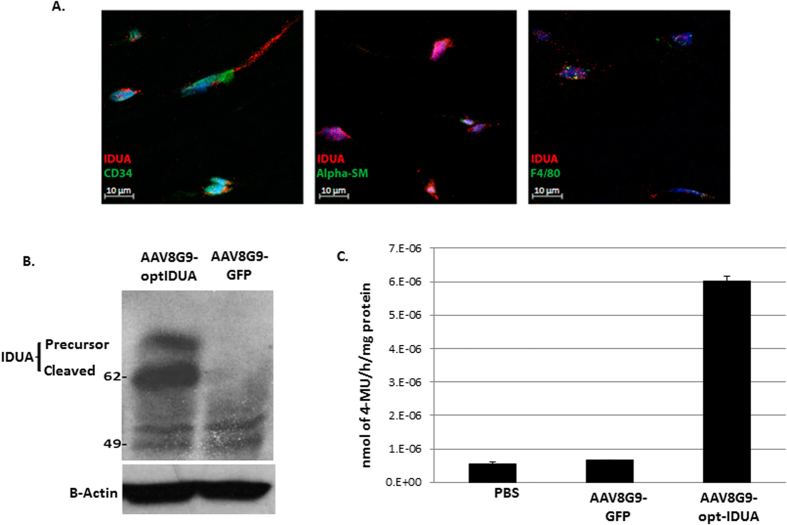
Restoration of IDUA activity in human corneas by AAV8G9-opt-IDUA. (**A**) A representative section of a normal non-injected cornea stained with IDUA antibody (red) and a cell marker (green). Hoechst was used for nuclei counterstain. The scale bar is equal to 10 μm. (**B**) Western blot detecting IDUA amounts produced after injection of AAV8G9-opt-IDUA. Administration of AAV8G9-GFP served as a vector infection control. B-actin serves as a loading control. (**C**) Functional activity of IDUA protein obtained from human corneas 7 days post-injection of AAV8G9-opt-IDUA. AAV8G9-GFP served as the negative control. The nmoles of 4-MU were normalized to one hour reaction and mg total protein. PBS corresponds to a human cornea injected with only the vehicle control. The experiment was performed in triplicate.

Following confirmation that the AAV8G9 capsid was the most efficient for corneal stroma transduction, we determined if the cell types transduced by AAV8G9 naturally produce IDUA. To do this, dual staining for the cell type marker and the IDUA protein was performed in normal untreated human corneas. The results indicate that all of the confirmed corneal cell types transduced by AAV8G9 naturally produce IDUA (Fig. 3A).

Next, AAV8G9-opt-IDUA preclinical vector preparations were produced by the UNC Vector Core and evaluated in normal human corneas *ex vivo*. For these experiments, injection of single-strand AAV8G9-GFP was evaluated and PBS served as the negative control. Seven days post-injection (1e^10^ vg in 50 μl), total protein was recovered and IDUA abundance was investigated by Western blotting. In whole corneal lysates, it was observed that the resting levels of IDUA are relatively low and that vector-derived IDUA, both the precursor and cleaved forms, was readily detected (Fig. 3B). Consistently, a 10-fold elevation of IDUA activity was observed in the AAV8G9-opt-IDUA injected corneas when compared to the normal levels found in human corneas (Fig. 3C).

**Figure 4:**
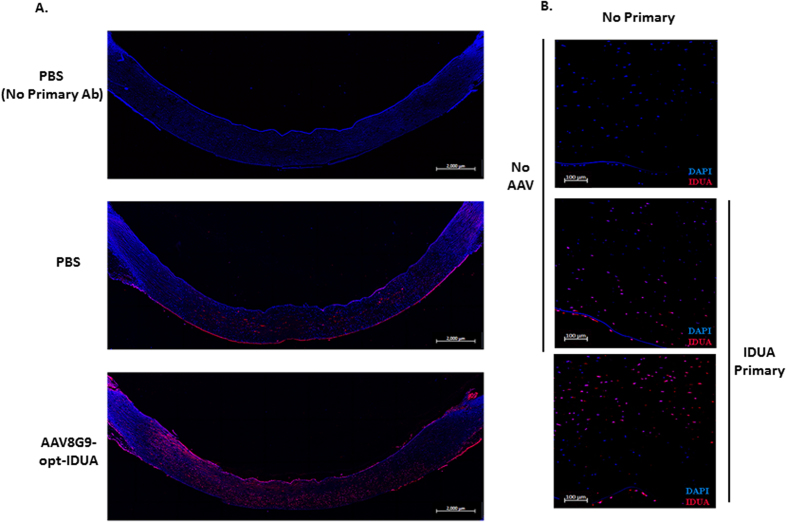
Distribution of IDUA protein following AAV-8G9-opt-IDUA transduction. (**A**) Immunofluorescence image showing the distribution of IDUA protein across a human cornea section seven days post-injection of AAV8G9-opt-IDUA or PBS (vehicle). The images were obtained with 10× objective and assembled by stitch processing. The scale bar is equal to 2000 μm. Hoechst was used for nuclei counterstain. (**B**) Cross section of the same stained human corneas but taken with a 20× objective. The top figure corresponds to the negative control of staining, which was treated in the same manner as the other samples but without the primary antibody. Hoechst was used for nuclei counterstain. The scale bar is equal to 100 μm. The experiment was performed in duplicate.

By immunofluorescence staining and detection on human cornea cross sections, the resting levels and distribution of IDUA were determined (Fig. 4). As MPS1 patient corneas are rare, staining under the same conditions but without the IDUA primary antibody served as the negative control. The results demonstrate relatively low levels of IDUA within the corneal stroma that were also present in the corneal epithelia and to a lesser extent, in the endothelial cell layer (Fig. 4). IDUA staining was also performed on human cornea cross sections that were injected with AAV8G9-opt-IDUA. The results correlate to those of the IDUA Western blot and the functional assay (Fig. 3) in that there is an approximate 5–10 fold increase in IDUA in normal human corneas treated with AAV8G9-opt-IDUA compared to the resting level in human corneas (Fig. 4). Furthermore, similar to the results observed with AAV8G9-GFP, vector derived IDUA appeared well distributed (Figs. 2C and 4).

**Figure 5:**
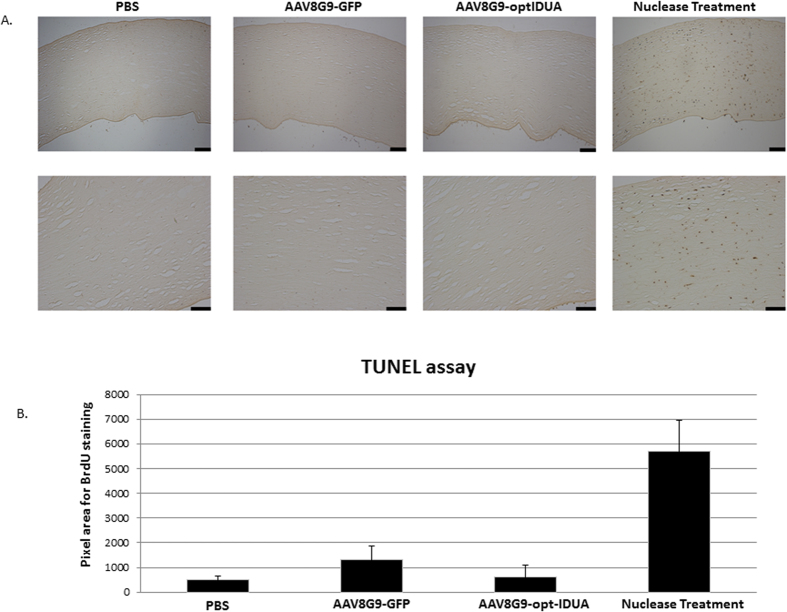
No apoptosis following AAV8G9-opt-IDUA injection in human corneas. (**A**) Human corneas injected with PBS, AAV8G9-GFP, or AAV8G9-opt-IDUA were processed 7 days later for Tunel staining. Top, images taken at 10×. Bottom, images taken at 20×. As a positive control, human corneas injected with PBS where nuclease treated. The scale bar is equal to 100 μm. (**B**) Quantitation of pixel area for total staining performed in (**A**). A minimum of 7 areas per cornea for each treatment were counted using SigmaScan Pro software. No statistical difference between cytotoxicity for corneas injected with AAV8G9-GFP or AAV8G9-opt-IDUA was observed (p > 0.05).

Considering restoration of IDUA to the MPS1 patient cornea, it is critical to determine any vector or IDUA related toxicity. Due to unavailability of a small animal model for MPS1 that develops cornea clouding, we performed apoptosis experiments in normal human corneas following AAV8G9-GFP or AAV8G9-opt-IDUA stromal injection (1e^10^ vg). Tunel staining, which detects fragmented DNA indicative of cellular apoptosis, was performed on day 7 post-injection. Although the nuclease-treated positive control samples demonstrated extensive Tunel staining, the staining of corneas in which IDUA abundance/function were significantly elevated did not show a significant difference to the ones non-injected or injected with AAV8G9-GFP (Fig. 5). These results are consistent with those obtained using MPS1 patient fibroblasts and collectively suggest the safety of AAV8G9-opt-IDUA gene therapy in MPS1 patient corneas. However, a complete toxicity study should be performed in an *in vivo* MPS1 model, where immune responses and/or liver toxicity could be evidenced.

## Discussion

Hematopoietic stem cell transplantation, after myeloablative chemotherapy using allogeneic bone marrow or umbilical cord blood donors has been shown to extend life and improve its overall quality, particularly when performed in MPS1 children under 2 years of age^18^,^19^,^20^,^21^,^22^. Engrafted donor cells provide a source of systemic IDUA and, through engraftment of donor microglial cells, enzyme replacement in the brain. Transplanted children, surviving 1–2 decades after HSCT with full donor chimerism, have normal blood IDUA levels with normal, to near normal, cognitive and cardiac functions. Later, and ongoing, manifestations of MPS1 after transplantation do occur and generally are limited to the joints, bones, and the eye, all of which are organs with lower profusion and lower delivery of donor-derived IDUA. As such, our preclinical corneal approach explored herein, was designed as a supplemental MPS1 therapy to address the shortcomings of stem cell transplantation and AAV gene therapy targeting the CNS.

As MPS1 patient corneas are rarely available, we initially investigated disease correction in MPS1 patient fibroblasts. The data demonstrate restoration of IDUA function using a low vector dose. This is, in part, due to the relatively low level of IDUA in normal fibroblasts which is presumably sufficient for normal cellular physiology (Fig. 1). Consistently, the abundance of IDUA found in normal human corneas was also modest. In fact, in whole cornea lysates, IDUA abundance by Western blotting was not readily detected, however, tissue histology identified resting levels of IDUA in corneal epithelia, endothelia and the majority in multiple cell types found in the stroma (Figs 3 and 4). This evaluation of IDUA abundance and AAV-mediated restoration highlights a very important aspect to our corneal gene therapy approach: mainly that only a low amount of IDUA is necessary to restore WT IDUA function in the corneas of MPS1 children. This evidence is supported by studies with the MPS1 canine model, in which cornea clouding was significantly improved in presence of low levels of IDUA , meanwhile higher levels of IDUA did not provide further benefit^23^. In our experiments, our chosen AAV dose in the cornea explants (1e^10^ vg) was found to be excessive and resulted in a 10-fold supraphysiological elevation of IDUA function. However, even at this high IDUA level no apoptosis was detected (Figs 3–5). Given the ability of secreted IDUA to cross-correct neighboring cells, the low resting levels in normal human corneas, and the efficiency of AAV8G9-opt-IDUA for human cornea transduction, the data herein suggest that a low vector dose will result in normal levels of IDUA in MPS1 patient corneas. Furthermore, reported complications associated with a neutralizing antibody response to the IDUA transgene product (IDUA) are not anticipated as there are no antibodies in the human cornea^24^. However, at this point we are not able to predict the occurrence of any type of immune reaction or liver-associated toxicity.

Given the collective data herein, restoring IDUA function in MPS1 corneas seems quite feasible. Furthermore, it appears that AAV8G9 elicits IDUA production in stromal cells that naturally produce IDUA, as well as in the endothelial layer (Fig. 4). Despite the ability to restore IDUA in the cornea, the question of whether or not restoration of IDUA in the cornea will reverse corneal clouding remains unanswered. However, several lines of evidence suggest that IDUA in the MPS1 cornea will reverse the corneal blindness^7^,^23^. In particular, the reduction of corneal opacity, but not complete correction, was reported in MPS1 patients receiving HSCT^5^. In that work it was hypothesized that the partial correction was due to restoration of IDUA in the corneal endothelium but not in the stroma. As our AAV-opt-IDUA strategy is capable of transducing both of these compartments (Fig. 4), the likelihood of reversing or preventing MPS1-associated corneal blindness, as a supplemental therapy to HSCT or AAV systemic gene delivery remains a viable possibility.

## Materials and Methods

### Production of AAV vectors

For cell culture experiments, a previously described triple transfection method was used to generate the vectors used herein^25^. This method used the pXR2 or pXR8G9 (manuscript submitted) plasmids, which all contain *rep2* of AAV and individually the capsid genes of the indicated serotype. The plasmid containing the intended AAV genome was first constructed by substituting the *egfp* gene in pTR-CMV-eGFP with the codon optimized IDUA cDNA (provided by GenScript) at the AgeI and SalI sites (now called pTR-CMV-opt-IDUA), or the wild type full-length hIDUA cDNA (kindly provided by Dr. Aronovich^26^) (now called pTR-CMV-_WT_IDUA). Following AAV production and cesium chloride gradient separation^25^, peak fractions were dialyzed against PBS, and titered by quantitative PCR (CMV_F CAA GTA CGC CCC CTA TTG AC, CMV_R AAG TCC CGT TGA TTT TGG TG) which was confirmed by Southern dot blot^25^. For the experiments in human explants, GMP grade vector preparations were provided by the UNC vector core.

### Cell culture and vector transduction

Human embryonic kidney 293 cells, MPS1 patient fibroblasts, and normal human fibroblasts^17^ were maintained at 37 °C in a 5% CO_2_ atmosphere in Dulbecco’s modified Eagle’s medium (Sigma) supplemented with 10% fetal bovine serum and penicillin–streptomycin (100 U/ml). Transduction of cultured cells was performed in a 24 well plate in a fixed volume. For these experiments, vector was added to the wells at the indicated viral genome per cell dose and was not removed for the duration of the experiment.

### Human Cornea Experiments

All experimental protocols were approved by the University of North Carolina at Chapel Hill. Human corneas were provided by the miracles in sight tissue procurement bank. These unidentified post-mortem tissue experiments were performed in accordance with the human subjects research office of human research ethics at the University of North Carolina at Chapel Hill (IRB-14-1019). The corneas of both genders were maintained in the supplied opti-mem at 37 °C in a 5% CO_2_ atmosphere. Injections were performed with a 31 gauge insulin syringe. India ink was used as a dye to verify the injections. Corneas were then cultured for 7 days and analyzed as described in the text. For detection of GFP by Elisa, human corneas were trimmed into several pieces of similar size and each piece was incubated in a separate well in a certain condition.

### Quantitative GFP detection by ELISA

Human cornea pieces were infected with 1 × 10^10^ viral genomes of each serotype: 8, 9, or the 8/9 chimeric 8G9 for seven days. Cell lysates were obtained by cutting each cornea into pieces and incubating the pieces with 200 μl M-PER® Mammalian Protein Extraction Reagent (Pierce, catalog number: 78503) and 2 μl Halt™ Protease Inhibitor Cocktail for 10 minutes, followed by repeating freezing and thawing procedures. An aliquot of 100 μl of each lysate was incubated at 4 °C overnight with the anti-GFP antibody coated plate (Cell Biolabs, Inc, catalog number AKR-121). Following extensive washes, a second incubation with an anti-GFP antibody, but this time biotinylated, was performed for 2 hours at room temperature. Wells were washed and streptavidin-enzyme conjugate was added to each well. The reaction took place by adding the substrate for the enzyme and once the color was developed, the reaction was stopped and absorbance at 450 nm was measured.

### Functional IDUA assay

Quantitative IDUA enzyme activities were measured as previously described^27^. Briefly, 10 μl of a supernatant or cell lysis solution, obtained the same way as for the detection of GFP by ELISA, were incubated with 50 μM 4-methylumbelliferyl alpha-L-iduronide made in 0.4 M sodium formate buffer, pH 3.5, containing 0.2% Triton x-100 at 37C for 60 min in the dark. Reactions were stopped by adding 80 μl of 0.5 M NaOH/glycine buffer pH 10.3. The 96 well plates were centrifuged for 1 min 13000 rpm at 4C and supernantans were transfered to a black 96w plate, clear bottom with lid for measuring fluorescence at 450 nm, following excitation at 365 nm. The amount of cleaved substrate was calculated from a standard curve, previously established with 4-methylumbelliferone and expressed as nmol/h/mg for enzyme activity in cell lysates, or nmol/h/10 μl for analysis of supernatants . The protein amount for each sample was determined with the BCA assay (Pierce™ BCA Protein Assay Kit, catalog number: 23225).

### Tunel Assay

Apoptotic cells generated due to the cellular cytotoxicity during AAV infection of human cornea explants were determined by deoxynucleotidyltransferase (TdT)-mediated dUTP-biotin nick end labeling with DAB. Tunel assay was performed with TACS2 TdT-Blue Label *in situ* apoptosis detection kit (Trevigen, Gaithersburg, Md.). Briefly, following deparaffinization and rehydration, the human cornea sections were digested for 20 min in proteinase K. Slides were washed with 1 × PBS and the endogenous peroxidase activity was quenched by incubating the tissues with 3% H_2_O_2_ solution in Methanol. After washing the slides with 1 × PBS, tissue sections were incubated with 1× TdT labeling buffer for 5 min. The labeling reaction consisting of incubating the cornea tissues with TdT enzyme, dNTPs with biotinylated dUTP, and 1 × manganese chloride in 1 × TdT labeling buffer for 1 h in a humidified chamber at 37C. The reaction was completed by incubating with 1 × TdT stop buffer. The fragmented DNA was visualized by treating the sections with streptavidin-conjugated horseradish peroxidase and DAB solution.

### Immunofluorescence Staining

Human cornea tissues were embedded in paraffin and sectioned at a thickness of 5 μm using a microtome. In order to stain the slide with immunofluorescence antibodies, sections were deparaffinized by incubating the slides in xylene for 5 min, two times total, followed by rehydration by immersing them sequentially in 100%, 95% and 70% ethanol solutions, 5 min each, and finally in water for 5 min. Before staining the sections, antigen retrieval procedure was performed in order to guarantee the exposure of epitopes, which were previously masked due to the paraffin embedding process. Specifically, slides were immerse into pre-heated antigen retrieval solution (Dako) at 98C for 7 min. Non-specific binding sites within the tissues were then blocked by incubating the slides with 10% NGS, in 1× PBS for 1 hour at RT. Slides were washed twice for 5 minutes each with 2% NGS in 1× PBS. Sections were then incubated overnight at 4 °C with primary antibody diluted appropriately in a solution consisting of 2% NGS in 1× PBS. After incubation with primary antibody, slides were washed three times, for 5 minutes each, in order to remove non-specifically bound primary antibody. The washing solution contained 2% NGS in 1× PBS. An appropriate fluorescently-labeled secondary antibody (5 μg/ml) was then added to the slides diluted in 2% NGS in 1× PBS, and slides were incubated for 1 hour at 4 °C. Finally, slides were washed twice with 2% NGS in 1× PBS three times, for five minutes each, followed by a last wash with 1× PBS at 4 °C. A couple of drops of Hoechst (Molecular probes- Life technologies, H3569 Hoechst 33258, Pentahydrate-1 μg/mL) were added for 7 minutes to counterstain the nuclei within the section, followed by a wash with water. Slides were then coverslipped with Cytoseal 60 (Thermo Fisher Scientific (NYSE: TMO)).

### Primary antibodies used for this study

For IDUA staining, Rabbit Polyclonal IDUA antibody (Biorbyt, catalog number: orb157615, dilution 1/50); for GFP staining, Chicken anti GFP antibody (Aves, catalog number: GFP-1020, dilution 1/100); for CD34 staining, mouse monoclonal antibody (clone: B-6) (Santa Cruz, sc-74499, dilution 1/100)), for α-Smooth Muscle Actin staining, mouse monoclonal antibody (R&D, clone #1A4, catalog number MAB1420, dilution 1/100), and for F4/80 marker staining, rat monoclonal antibody, (clone: BM8) (Santa Cruz, sc-52664, dilution 1/100). Secondary antibodies were the following: Alexa Fluor® 594 goat anti-rabbit IgG (A-11012) (Gibco – Invitrogen, Carlsbad, CA), Alexa Fluor® 594 goat anti-chicken IgG (A-11039) (Gibco – Invitrogen, Carlsbad, CA), Alexa Fluor® 594 goat anti-rat IgG (A-11006) (Gibco – Invitrogen, Carlsbad, CA) and Alexa Fluor® 488 goat anti-mouse IgG (A-11001) (Gibco – Invitrogen, Carlsbad, CA). Images from each slide were taken using a Zeiss LSM 780 Confocal Microscope with a 40× objective (Olympus, Tokyo, Japan). Images that comprised the complete human cornea were taken with 10× objective, the title function and then stitched. Images were then processed using Adobe Photoshop. Sections from each tissue were stained with secondary antibody alone as negative control staining in all experiments.

### MPS1 Cell Transfection and Western Blot

MPS1 fibroblast patient cells were plated onto a 24 well plate at 20,000 cells per well. After 24 hours, nine wells were transfected for each treatment adding a mix 1ug of plasmid DNA, 3μl of PEI and 60 μl of DMEM to each well. 24 hours after transfection, 40μl of supernatant were collected from each well and 3 wells were combined to form each sample. 40 μl of DMEM were added to each well to maintain the same volume for later supernatant collections. 48 hours after transfection, total supernatant was collected combining 3 wells to again form triplicates. At that time total cell protein was harvested by adding 70 μl of Mammalian Protein extraction Reagent (Thermo Scientific Cat: 78501), per well and following reagent protocol.

For Western blot, Protein lysate was added to a solution of 5% beta-mercapto-ethanol in 4× Nupage sample buffer. The resulting solution was boiled for 10 minutes and chilled on ice for 10 minutes and then run in a 10% Bis-tris pre-cast gel. Gel was run in 1× MOPS running buffer, and transferred to a nitrocellulose membrane. The membrane was blocked with 5% milk in ddH20, and probed with mouse host IDUA antibody (R&D Systems MAB-4119) for 2 hours at 1:500 dilution in PBS-Tween solution (0.5% Tween 20 in 1 × PBS) or mouse host beta-actin antibody (Sigma) at 1:5000 dilution in PBS-Tween. This was followed by mouse horse radish peroxide secondary antibody at 1:10000 dilution in PBS-Tween. Western-Bright Sirius chemi-luminescence reagent was used according to product protocol and blots were exposed using autoradiography film.

### Cell Transfection comparison of wild-type against codon optimized IDUA

293 cells were plated onto a 24 well plate at 100,000 cells per well. After 24 hours, 3 wells were transfected using a mix of 1ug of plasmid DNA, 3 μl of PEI and 60 μl of DMEM per well. Total cell protein was harvested after 72 hours by adding 70 μl of Mammalian Protein extraction Reagent (Thermo Scientific Cat: 78501), to each well after saving the cell culture supernatant.

### MPS1 Patient Cell Toxicity Assay

Patient Cells were plated onto a 24 well plate at 20,000 cells per well. AAV2-opt-IDUA was added after 24 hours in treatments of 5,000 and 50,000 viral genomes per cell with a total of eight replicates per treatment. After 72 hours, each well’s supernatant was saved. This was followed by cell resuspension using 150 μl of 0.05% trypsin. Trypsin was deactivated by adding back the respective supernatant to each well. All samples were analyzed using a Beckman Vi-cell XR cell viability dye exclusion analyzer.

## Additional Information

**How to cite this article**: Vance, M. *et al*. AAV Gene Therapy for MPS1-associated Corneal Blindness. *Sci. Rep*. **6**, 22131; doi: 10.1038/srep22131 (2016).

## Supplementary Material

Supplementary Information

## References

[b1] Aldenhoven M. . Long-term outcome of Hurler syndrome patients after hematopoietic cell transplantation: an international multicenter study. Blood 125(13), p. 2164–72 (2015). 25624320 10.1182/blood-2014-11-608075

[b2] Janson C. G. . Comparison of Endovascular and Intraventricular Gene Therapy With Adeno-Associated Virus-alpha-L-Iduronidase for Hurler Disease. Neurosurgery 74(1), p. 99–111 (2014). 24077583 10.1227/NEU.0000000000000157 PMC4116107

[b3] Wolf D. A. . Direct gene transfer to the CNS prevents emergence of neurologic disease in a murine model of mucopolysaccharidosis type I. Neurobiol Dis 43(1), p. 123–33 (2011). 21397026 10.1016/j.nbd.2011.02.015 PMC4416491

[b4] Hinderer C. . Intrathecal gene therapy corrects CNS pathology in a feline model of mucopolysaccharidosis I. Mol Ther 22(12), p. 2018–27 (2014). 25027660 10.1038/mt.2014.135 PMC4429692

[b5] Huang Y. . Ultrastructural study of the cornea in a bone marrow-transplanted Hurler syndrome patient. Exp Eye Res 62(4), p. 377–87 (1996). 8795456 10.1006/exer.1996.0043

[b6] Alroy J., Haskins M. & Birk D. E., Altered corneal stromal matrix organization is associated with mucopolysaccharidosis I, III and VI. Exp Eye Res 68(5), p. 523–30 (1999). 10328965 10.1006/exer.1998.0622

[b7] Fahnehjelm K. T. . Ocular findings in four children with mucopolysaccharidosis I-Hurler (MPS I-H) treated early with haematopoietic stem cell transplantation. Acta Ophthalmol Scand 84(6), p. 781–5 (2006). 17083538 10.1111/j.1600-0420.2006.00743.x

[b8] Hobbs J. R., Bone marrow transplantation for inborn errors. Lancet 2(8249), p. 735–9 (1981). 6116868 10.1016/s0140-6736(81)91059-x

[b9] Hoogerbrugge P. M. . Allogeneic bone marrow transplantation for lysosomal storage diseases. The European Group for Bone Marrow Transplantation. Lancet 345(8962), p. 1398–402 (1995). 7760610 10.1016/s0140-6736(95)92597-x

[b10] Vellodi A. . Bone marrow transplantation for mucopolysaccharidosis type I: experience of two British centres. Arch Dis Child 76(2), p. 92–9 (1997). 9068295 10.1136/adc.76.2.92 PMC1717089

[b11] Gullingsrud E. O., Krivit W. & Summers C. G., Ocular abnormalities in the mucopolysaccharidoses after bone marrow transplantation. Longer follow-up. Ophthalmology 105(6), p. 1099–105 (1998). 9627663 10.1016/S0161-6420(98)96014-6

[b12] Souillet G. . Outcome of 27 patients with Hurler’s syndrome transplanted from either related or unrelated haematopoietic stem cell sources. Bone Marrow Transplant 31(12), p. 1105–17 (2003). 12796790 10.1038/sj.bmt.1704105

[b13] Sharma A. . AAV serotype influences gene transfer in corneal stroma *in vivo*. Exp Eye Res 91(3), p. 440–8 (2010). 20599959 10.1016/j.exer.2010.06.020 PMC2926174

[b14] Hippert C. . Corneal transduction by intra-stromal injection of AAV vectors *in vivo* in the mouse and *ex vivo* in human explants. PLoS One 7(4), p. e35318 (2012). 22523585 10.1371/journal.pone.0035318 PMC3327666

[b15] Mohan R. R. . Targeted decorin gene therapy delivered with adeno-associated virus effectively retards corneal neovascularization *in vivo*. PLoS One 6(10), p. e26432 (2011). 22039486 10.1371/journal.pone.0026432 PMC3198476

[b16] Grieger J. C. & Samulski R. J., Adeno-associated virus as a gene therapy vector: vector development, production and clinical applications. Adv Biochem Eng Biotechnol 99, p. 119–45 (2005). 16568890

[b17] Simpson D. A. . Telomerase expression is sufficient for chromosomal integrity in cells lacking p53 dependent G1 checkpoint function. J Carcinog 4, p. 18 (2005). 16209708 10.1186/1477-3163-4-18 PMC1262734

[b18] Shapiro E. G. . Neuropsychological outcomes of several storage diseases with and without bone marrow transplantation. J Inherit Metab Dis 18(4), p. 413–29 (1995). 7494400 10.1007/BF00710053

[b19] Whitley C. B. . Long-term outcome of Hurler syndrome following bone marrow transplantation. Am J Med Genet 46(2), p. 209–18 (1993). 8484412 10.1002/ajmg.1320460222

[b20] Prasad V. K. . Unrelated donor umbilical cord blood transplantation for inherited metabolic disorders in 159 pediatric patients from a single center: influence of cellular composition of the graft on transplantation outcomes. Blood 112(7), p. 2979–89 (2008). 18587012 10.1182/blood-2008-03-140830 PMC2556628

[b21] Boelens J. J. . Outcomes of transplantation of unrelated cord blood in children with malignant and non-malignant diseases: an Utrecht-Prague collaborative study. Bone Marrow Transplant 43(8), p. 655–7 (2009). 18997829 10.1038/bmt.2008.367

[b22] Summers C. G. . Ocular changes in the mucopolysaccharidoses after bone marrow transplantation. A preliminary report. Ophthalmology 96(7), p. 977–84; discussion 984–5 (1989). 2505207 10.1016/s0161-6420(89)32795-3

[b23] Traas A. M. . Correction of clinical manifestations of canine mucopolysaccharidosis I with neonatal retroviral vector gene therapy. Mol Ther 15(8), p. 1423–31 (2007). 17519893 10.1038/sj.mt.6300201

[b24] Hinderer C. . Neonatal Systemic AAV Induces Tolerance to CNS Gene Therapy in MPS I Dogs and Nonhuman Primates. Mol Ther 23(8), p. 1298–307 (2015). 26022732 10.1038/mt.2015.99 PMC4817868

[b25] Grieger J. C., Choi V. W. & Samulski R. J., Production and characterization of adeno-associated viral vectors. Nat Protoc 1(3), p. 1412–28 (2006). 17406430 10.1038/nprot.2006.207

[b26] Aronovich E. L. . Systemic correction of storage disease in MPS I NOD/SCID mice using the sleeping beauty transposon system. Mol Ther 17(7), p. 1136–44 (2009). 19384290 10.1038/mt.2009.87 PMC2835207

[b27] Garcia-Rivera M. F. . Characterization of an immunodeficient mouse model of mucopolysaccharidosis type I suitable for preclinical testing of human stem cell and gene therapy. Brain Res Bull 74(6), p. 429–38 (2007). 17920451 10.1016/j.brainresbull.2007.07.018 PMC2148227

