# Supplementary material for: AAV Gene Therapy for MPS1-associated Corneal Blindness
Source: Sci Rep. 2016 Feb 22;6:22131. doi: 10.1038/srep22131 (PMC4761992; doi:10.1038/srep22131)
Supplement: Supplementary Information [file srep22131-s1.pdf]

Supplementary Information

AAV Gene Therapy for MPS1-associated Corneal Blindness

Melisa Vance*[1] , Telmo Llanga*[1, 2], Will Bennett[1, 2], Kenton Woodard[1] , Giridhar Murlidharan[1, 3], Neil Chungfat[2], Aravind Asokan[1,3], Brian Gilger[5], Joanne Kurtzberg[6], R. Jude Samulski[1, 4], Matthew L Hirsch[1, 2].

[1]Gene Therapy Center, University of North Carolina at Chapel Hill, NC, 27599
[2]Department of Ophthalmology, University of North Carolina, Chapel Hill, NC, 27599
[3]Department of Genetics, University of North Carolina, Chapel Hill, NC, 27599
[4]Department of Pharmacology, University of North Carolina, Chapel Hill, NC, 27599
[5]College of Veterinary Medicine, NCSU-CVM, Clinical Sciences, Raleigh, NC, USA
[6]Department of Pediatrics, Duke University, Durham, NC, 27710

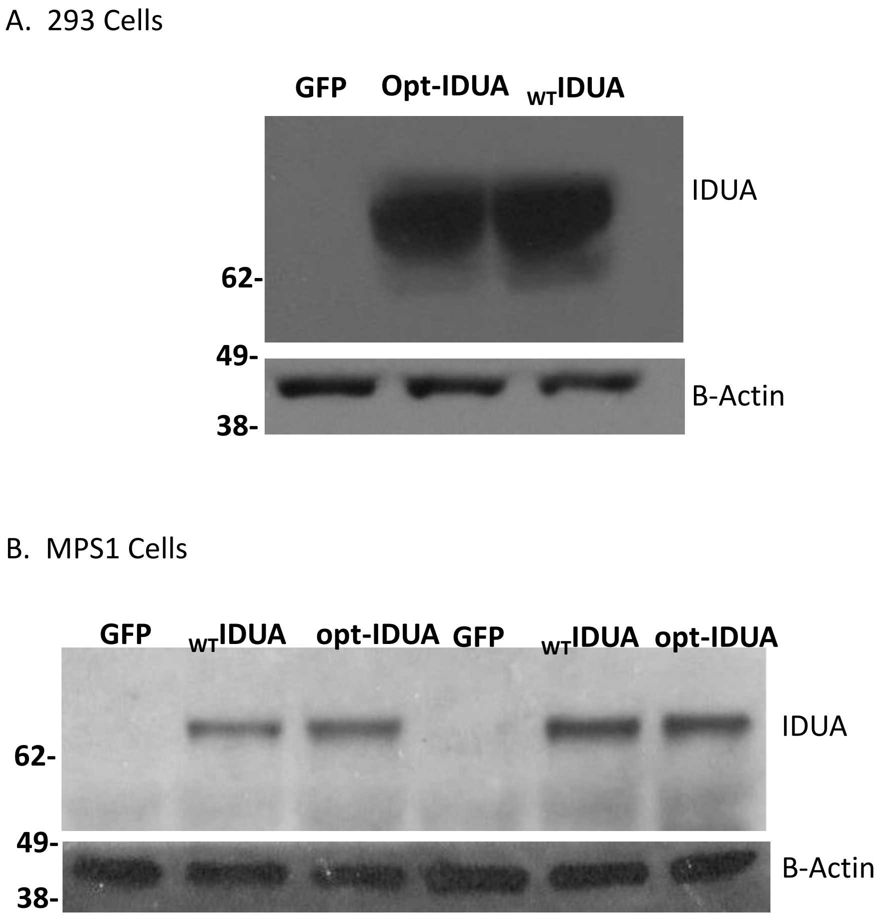

Figure S1. Evaluation of Optimized IDUA. 293 cells (A) or MPS1 human fibroblasts (B) were transfected with identical expression cassettes containing the wild type ($_{WT}$IDUA) or codon-optimized (opt-IDUA) IDUA open reading frames. Three days post-transfection Western blot analysis was used to determine the overall abundance of IDUA and B-actin. The sizes of a molecular weight marker is listed in kiloDaltons on the left.

Fig S1

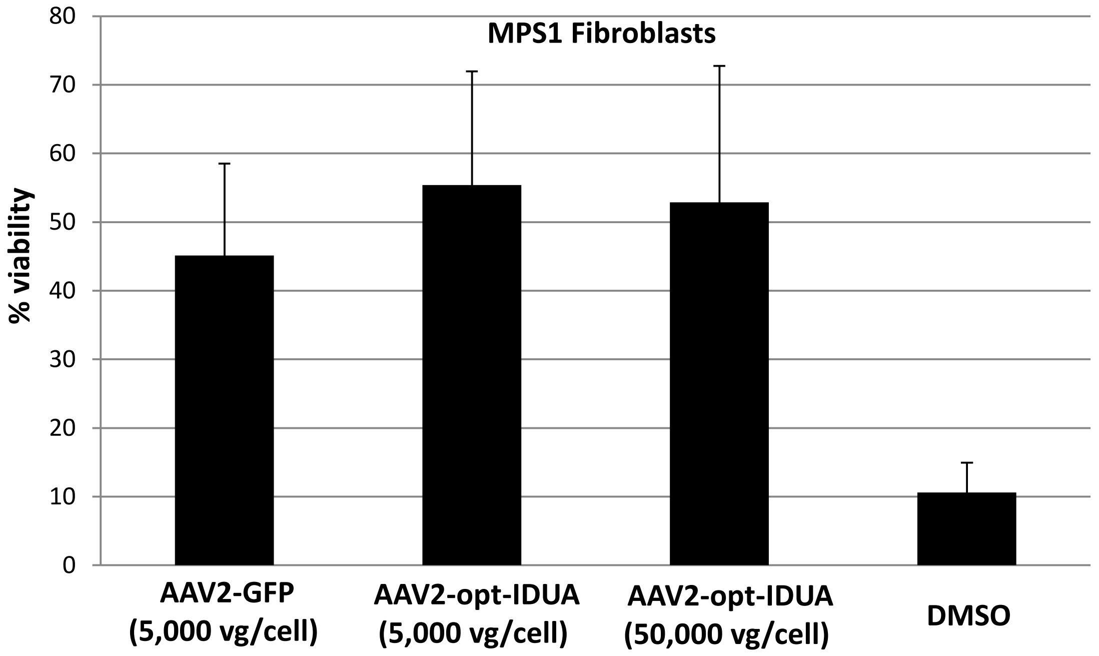

Figure S2. Analysis of AAV2-optIDUA toxicity in MPS1 patient fibroblasts. Trypan blue dye exclusion assay was performed on patient fibroblast cells following infection with AAV2 vector carrying opt-IDUA sequence or GFP reporter gene. Incubation with DMSO was used as a cell death control. Vg, viral genomes.

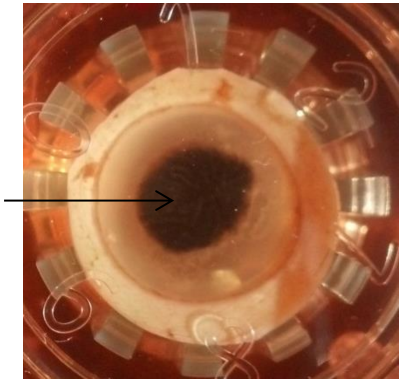

Figure S3. Intrastromal injection. Representative image demonstrating the dye distribution following an intrastromal vector injection in a cornea explant. India ink was used in a vector prep with a final volume of 50 µl for each injection.
